# Lambda-cyhalothrin-induced pancreatic toxicity in adult albino rats

**DOI:** 10.1038/s41598-023-38661-1

**Published:** 2023-07-18

**Authors:** Samar Sakr, Walaa A. Rashad

**Affiliations:** 1 Department of Forensic Medicine and Clinical Toxicology, Faculty of Medicine, Zagazig University, Zagazig, Egypt; 2 Department of Anatomy and Embryology, Faculty of Medicine, Zagazig University, Zagazig, Egypt

**Keywords:** Biochemistry, Environmental sciences, Anatomy, Endocrinology, Medical research

## Abstract

Lambda-cyhalothrin (LCT) is one of the most frequently utilized pyrethroids. This study aimed to explore the toxic effects of subacute exposure to LCT on the pancreas and the hepatic glucose metabolism in adult male albino rats. 20 rats were equally grouped into; Control group and LCT group. The latter received LCT (61.2 mg/kg b.wt.), orally on a daily basis for 28 days. At the end of experiment, blood samples were collected for the determination of serum glucose and insulin levels. Pancreases were harvested and levels of malondialdehyde (MDA); catalase (CAT); superoxide dismutase (SOD); reduced glutathione (GSH); tumor necrosis factor-α (TNF-α); interleukin-6 (IL-6); nuclear factor erythroid 2–related factor 2 (Nrf2); heme oxygenase 1 (HO-1); and nuclear factor kappa-light-chain-enhancer of activated B cells (NF-κB) were assessed. Also, liver samples were analyzed for the activity of glucose metabolism enzymes, glycogen content, and pyruvate and lactate concentrations. Histopathological and immunohistochemical examinations of pancreatic tissues were undertaken as well. Results revealed hyperglycemia, hypoinsulinemia, increased MDA, TNF-α, IL-6, and NF-κB levels, in association with reduced CAT, SOD, GSH, Nrf2, and HO-1 levels in LCT group. Liver analyses demonstrated a clear disturbance in the hepatic enzymes of glucose metabolism, diminished glycogen content, decreased pyruvate, and increased lactate concentrations. Besides, pancreatic islets displayed degenerative changes and β-cells loss. Immunohistochemistry revealed diminished area percentage (%) of insulin and Nrf2 and increased TNF-α immunoreaction. In conclusion, subacute exposure to LCT induces pancreatic toxicity, mostly via oxidative and inflammatory mechanisms, and dysregulates hepatic glucose metabolism in albino rats.

## Introduction

Pyrethroids are globally among the most commonly used insecticides. Despite the claim of low toxicity to the non-target organisms, the extensive usage of pyrethroids has augmented the risk of human exposure^1^.

Lambda-cyhalothrin (LCT: α-cyano-3-phenoxybenzyl-3-(2-chloro-3,3,3-trifluoro-1 propenyl)-2,2- dimethylcyclopropane carboxylate) is a type II pyrethroid^2^. It is widely employed inside and around houses, as adulticidal, ovicidal, and larvicidal insecticide for controlling Lepidoptera, Hemiptera, Diptera, Coleoptera, mosquitoes, flies, ants, and cockroaches^3^. Also, LCT is usually applied during the cotton plantation season to control pests’ infestation^4^.

Pyrethroids including LCT cause sustained neuronal discharge via stimulation of the sodium channels in nerve membranes^5^. The lipophilic nature of pyrethroids enhances their absorption and accumulation in fatty tissues^6^, and permits their direct access to the nervous system via the sensory organs of peripheral nerves^7^.

Previous studies have demonstrated the ability of LCT to enhance cytotoxicity^8^, inflammation^9^, developmental toxicity, and endocrinal disruption in vertebrates^10^. Similar to other pyrethroids, LCT tends to accumulate within the biological membranes^11^ causing oxidative stress injury, and cytokines mediated activation of vascular endothelium and inflammatory mediators^12^.

Diabetes mellitus (DM) is a worldwide metabolic disorder defined by the presence of hyperglycemia owing to impaired insulin secretion, defective insulin action, or both. Diabetic people have been estimated at 3% of the world population and are expected to be 6% in 2025^13^. Prolonged exposure to pyrethroids like cypermethrin (CYP)^14^, deltamethrin (DMT)^15^, and permethrin^16^ has been proved to disturb glucose metabolism, as indicated by increased blood glucose level and defective insulin secretion. Oxidative stress has been proposed as a key mechanism for such toxicity^15^.

The pancreas controls glucose homeostasis via insulin secretion^15^. Liver is an insulin-sensitive tissue that maintains the balance between glucose production and utilization. A partial or total loss of pancreatic insulin can disrupt glucose homeostasis via dysregulating the hepatic enzymes of glucose metabolism^17^.

Information on the interplay between pesticides and diabetes is relatively new. Many epidemiological studies have investigated the possible association between the development of diabetes and exposure to pesticides, like organochlorines and organophosphates. However, pyrethroids-relevant studies remain very limited^18^.

So, this work aimed to explore the toxic effects of subacute exposure to LCT on the pancreas as well as the hepatic glucose metabolism in adult male albino rats.

## Material and methods

### Chemicals

Lambda-cyhalothrin (LCT), CAS chemical name (α -cyano-3- phenoxybenzyl-3-(2-chloro- 3,3,3-trifluoro-1-propenyl)- 2,2 dimethyl cyclopropanecarboxylate, emulsified concrete (EC), was purchased under the commercial name of Lambda C 5% from Kafr El-Zayat for pesticides and chemical Company, Kafr El-Zayat City, Gharbia Governorate, Egypt.

### Experimental animals

This study was conducted in close conformity with the regulations of the Institutional Animal Care and Use Committee of Zagazig University (ZU-IACUC), approval number (ZU-IACUC/3/F/124/2021), and was carried out following the ARRIVE guidelines.

Before starting the experiment, 20 adult male albino rats were acclimatized for two weeks at room temperature (23 ± 2 °C), relative humidity (55 ± 5%), and normal light–dark cycles of 12 h. Rats had free ad libitum access to fresh tap water and the standard pellet diet containing all nutritive elements.

Rats (150–200 g; 16–18 weeks) were divided randomly into two equal groups; the control group received 1 mL distilled water/rat, daily via oral gavage, and the LCT-treated group which was orally gavaged with LCT at a dose of 61.2 mg/kg b.wt.^19^ dissolved in 1 mL distilled water (vehicle of LCT) for each rat, on a daily basis for 28 days. The selected dose represented 1/10 of LD_50_ of LCT^20^ and was chosen for being toxic but not lethal to the rats^8^. Every week, rats were weighed to maintain a comparable dose/kg body weight all through the experiment. Oral gavage was done through a metallic tube specific for rats.

### Specimen collection

After overnight fasting, animals were anesthetized using pentobarbital (50 mg/kg I.P). Afterthat, blood samples (3–5 mL) were collected by the cardiac puncture into a non-heparinized glass tube to allow clot formation. Then, samples were centrifuged (3000 × *g* for 10 min at 4 °C) to separate sera that were stored immediately at − 80 °C.

Subsequently, euthanasia of rats was done via cervical dislocation. Pancreases and livers were removed, thoroughly washed in ice-cold saline, inspected for gross anomalies, and then bisected into two portions. The first portion of pancreas was homogenized in ice-cold buffer (10 mM potassium phosphate) and centrifuged (4000 × *g* at 4 °C) for 10 min. Then, the supernatant was collected for various biochemical parameters. The second portion was fixed in 10% formalin solution, and processed for the histological and immunohistochemical examinations. For the liver, the first portion was homogenized in 20 times volume 0.1 M Tris–HCl for 10 min and centrifuged (10,000 × *g* at 4 °C) for 30 min. After that, the supernatant was obtained to measure the activity of glucose metabolism enzymes, and lactate and pyruvate concentrations. A second small portion of wet liver was snap-frozen in liquid nitrogen and kept at − 80 °C for glycogen analysis.

### Biochemistry

#### Serum glucose and insulin

Serum glucose level was determined colorimetrically using the kits of Bio-diagnostic Company, Egypt. Serum insulin was measured by the enzyme-linked immunosorbent assay (ELISA) kits (EIA-2048, 96 wells, DRG Instruments GmbH, Marburg, Germany).

#### Pancreatic oxidative parameters

Malondialdehyde (MDA) levels were detected as instructed by Ohkawa et al.^21^ using Elabscience kits (Cat. No. E-BC-K025-S, USA). The absorbance was assessed spectrophotometrically at 532 nm.

Catalase (CAT) was measured following Aebi’s method^22^, and kits of MyBioSource (Cat. NO. MBS701908, USA) were used. H2O2 decomposition was spectrophotometrically determined at 240 nm for 60 s, where the change in the absorbance was used as a measurement of CAT activity.

Superoxide dismutase (SOD) was determined by using MyBioSource kits (Cat No. MBS266897, USA) as described by Misra and Fridovich^23^. Alteration in the absorbance was spectrophotometrically noticed at 480 nm.

Reduced glutathione (GSH) was detected following Sedlak and Lindsay’s method^24^ by using the kits of MyoBioSource (Cat. No. MBS265966, USA). The reaction between the thiol groups and 5–5-dithiobis-(2-nitrobenzoic acid) resulted in the formation of a chromophore absorbing the light at 412 nm. GSH level was calculated using a standard reference curve.

#### Pancreatic pro-inflammatory cytokines

The determinations of the tumor necrosis factor-α (TNF-α) and interleukin-6 (IL-6) in the pancreatic tissues were done as described by the manufacturer using ELISA Kits of MyBioSource (TNF-α: Cat. NO. MBS282960; IL-6: Cat. NO. MBS355410, USA).

#### Pancreatic protein Levels of nuclear factor erythroid 2–related factor 2 (Nrf2), heme oxygenase 1 (HO-1), and nuclear factor kappa-light-chain-enhancer of activated B cells (NF-κB)

The pancreatic Nrf2, HO-1, and NF-κB protein levels were measured in the supernatant of pancreatic tissue homogenate. Commercial ELISA kits purchased from MyBioSource (Nrf2: Cat. No. MBS012148; NF-κB: Cat. No. MBS453975, USA) and Abcam (HO-1: Cat. No. ab213968, UK) were used in accordance with the manufacturer’s instructions. The optical density for each of Nrf2, HO-1, and NF-κB was read at 450 nm within 15 min using ELISA readers.

#### Hepatic enzymes of glucose metabolism

The hexokinase (HK; EC 2.7.1.1) activity was analyzed as described by DeWaal et al.^25^. The absorbance of samples was monitored at 340 nm.

The activity of glucose-6-phosphatase (G6Pase; EC3.1.3.9) was determined following Koide and Oda’s method^26^ with minor modification. Absorbance was detected at 340 nm and correlated to the liberated inorganic phosphate (Pi) units per hour per mg of tissue protein.

Lactate dehydrogenase (LDH; EC 1.1.1.27) activity was detected in the supernatant of hepatic homogenate according to Prameelamma and Swami^27^. The developed color of formazan was measured by a spectrophotometer at 495 nm against a toluene blank.

Glycogen synthase (GSase; E.C.2.4.2.11) activity was measured as described by Leloir and Goldemberg^28^. Uridine diphosphate glucose (UDP-glucose) represents the precursor for glycogen synthesis. So, results were correlated to the UDP formed per hour per mg of tissue protein and absorbance was monitored at 280 nm.

In line with Hers and Van Hoof^29^, the activity of glycogen phosphorylase (GPase; E.C.2.4.1.1) was determined colorimetrically at 680 nm as a read-out of liberated inorganic phosphate.

#### Hepatic glycogen

The glycogen was measured according to Murat and Serfaty^30^. The liver homogenate was combined with exo-1,4-α-glucosidase to produce glucose. Then, glucose levels were determined using a glucose assay kits (Bio-diagnostic Company, Egypt).

#### Hepatic lactate and pyruvate

Hepatic lactate and pyruvate concentrations were determined colorimetrically using L-lactate Assay kits (cat. no. ab65331) and Pyruvate Assay Kits (cat. no. ab65342) of Abcam, according to the manufacturer’s instructions.

### Histopathology

#### Hematoxylin and Eosin (H&E) and Masson’s trichrome

The harvested pancreases were sliced, processed, and embedded in paraffin blocks. After that, blocks were cut and stained with H&E and Masson trichrome stains^31^. Slides were examined by light microscopy (OLYMPUS, Japan).

#### Immunohistochemistrty (insulin, Nrf2, and TNF-α)

Immunohistochemical examinations were undertaken based on the streptavidin–biotin immunoperoxidase technique. Slides were incubated overnight with the primary antibodies including, anti-insulin rabbit monoclonal antibody (1: 50 dilution in PBS, Cat. No. ab4051, ABclonal, USA); anti Nrf2 rabbit polyclonal antibody (1:100 dilution, Cat. No. YAP1621, Biospes, China); and anti TNF-α rabbit polyclonal antibody (1: 50 dilution, Cat. No. A0277, ABclonal, USA). Slides were examined by light microscopy at the Department of Anatomy, Faculty of Medicine, Zagazig University.

#### Morphometric analysis

Estimation of the diameter of islets of Langerhans and area % of collagen fibers was carried out for H&E and Masson’s trichrome stained sections, respectively. Also, the area % of immunoreactivity to insulin, Nrf2, and TNF-α were determined at a magnification X 400 within 6 fields for each rat using g ImageJ IHC Profiler plugin ImageJ (Fiji Image J) software (National Institute of Health; NIH, Bethesda, MD, USA). Thereafter, results were presented as means ± standard deviations (SD).

### Statistics

Data were analysed using SPSS version 19 (SPSS Inc., Chicago, IL, USA). Results were presented as means ± SD. One-way ANOVA was used for comparing the two groups. A p-value of < 0.05 was considered as statistically significant.

### Ethical approval

The study was approved by the Institutional Animal Care and Use Committee, Zagazig University (ZU-IACUC/3/F/124/2021).

## Results

### Serum glucose and insulin

Results demonstrated a highly significant increase in the glucose levels by nearly 1.6-fold along with a 0.5-fold decrease in the serum insulin levels, in LCT group compared to the control (Table 1).

**Table 1:** Serum biochemistry of experimental groups.

Parameter	Treatment group (n = 10)		F- value of the one-way ANOVA
	Control	LCT	
Glucose (mg/dL)	88.98 ± 5.29	146.53 ± 6.19*	499.82
Insulin (μIU/mL)	11.19 ± 0.66	5.28 ± 0.26*	686.63

*LCT* lambda-cyhalothrin.

*p < 0.001 when values are compared to control group.

Values are expressed as mean ± SD.

n = number of rats per group.

### Pancreatic oxidative stress indices

Pancreatic MDA levels of LCT-treated rats were significantly increased by threefold while the levels of CAT, SOD, and GSH were significantly reduced by nearly 0.5-fold, fourfold, and 2.5-fold, respectively in the LCT group, in comparison with the control group (Table 2).

**Table 2:** Oxidative stress and inflammatory indices in pancreas of experimental groups.

Parameter	Treatment group (n = 10)		F- value of the one-way ANOVA
	Control	LCT	
MDA (nmol/g)	12.52 ± 0.74	38.69 ± 1.32*	2971.79
CAT (U/g)	0.69 ± 0.02	0.35 ± 0.01*	1820.08
SOD (U/g)	14.11 ± 0.84	3.62 ± 0.18*	1498.53
GSH (mg/g)	55.88 ± 3.32	22.09 ± 0.76*	985.16
TNF-α (pg/mg/ptn)	37.69 ± 4.19	171.72 ± 7.25*	2559.81
IL-6 (pg/mg/ptn)	131.81 ± 5.57	251.37 ± 9.19*	1238.81

*LCT* lambda-cyhalothrin, *MDA* malondialdehyde, *CAT* catalase, *SOD* superoxide dismutase, *GSH* reduced glutathione, *TNF-α* tumor necrosis factor-alpha, *IL-6* interleukine-6.

*p < 0.001 when values are compared to control group.

Values are expressed as mean ± SD.

n = number of rats per group.

### Pancreatic inflammatory parameters

The LCT-treated group revealed a highly significant increase in TNF-α by 4.5-fold and IL-6 by 1.9-fold, compared to the control group (Table 2).

### Pancreatic Nrf2, HO-1, and NF-κB protein levels

The Nrf2 and HO-1 protein levels were significantly reduced by 1.6-fold and 2.6-fold, respectively. Meanwhile, NF-κB levels were significantly increased by 1.8-fold in the LCT group when compared to the control (Table 3).

**Table 3:** Protein levels of Nrf-2, HO-1, and NF-kB in pancreas of experimental groups.

Parameter	Treatment group (n = 10)		F- value of the one-way ANOVA
	Control	LCT	
Nrf2 (pg/g)	89.99 ± 3.59	52.76 ± 2.23*	775.58
HO-1 (pg/g)	68.28 ± 4.06	25.57 ± 1.13*	1028.92
NF-κB (pg/mg/ptn)	121.76 ± 5.14	225.95 ± 9.54*	923.43

*LCT* lambda-cyhalothrin, *Nrf-2* nuclear factor erythroid 2–related factor 2, *HO-1* heme oxygenase 1, *NF-κB* nuclear factor kappa-light-chain-enhancer of activated B cells.

*p < 0.001 when values are compared to control group.

Values are expressed as mean ± SD.

n = number of rats per group.

### Hepatic enzymes of glucose metabolism

Results demonstrated a highly significant reduction in the hexokinase levels by 1.6-fold and glycogen synthase activity by 1.3-fold in the LCT group. Contrary, the activities of both of glucose-6-phosphatase and lactate dehydrogenase enzymes were significantly increased by 1.5-fold. Also, the glycogen phosphorylase activity demonstrated a 1.2-fold increase in comparison with the control group (Table 4).

**Table 4:** Hepatic enzymes of glucose metabolism in experimental groups.

Parameter	Treatment group (n = 10)		F- value of the one-way ANOVA
	Control	LCT	
Hexokinase μmoles of glucose-6-phosphate/h/mg of protein	257.02 ± 10.86	158.26 ± 5.69*	599.98
Glucose-6-phosphatase (μmoles of Pi liberated/h/mg of protein)	1000.90 ± 42.28	1524.13 ± 55.70*	1070.142
Lactate dehydrogenase (μmoles of pyruvate formed/h/mg of protein)	232.50 ± 9.82	368.97 ± 13.48*	669.16
Glycogen synthase (μ moles of UDP formed/h/mg protein)	810.44 ± 34.23	606.90 ± 22.18*	248.99
Glycogen phosphorylase (μmoles Pi liberated/h/mg protein)	610.39 ± 25.78	790.02 ± 28.87*	215.33

*LCT* lambda-cyhalothrin, *Pi* inorganic phosphate, *UDP* uridine diphosphate glucose.

*p < 0.001 when values are compared to control group.

Values are expressed as mean ± SD.

n = number of rats per group.

### Hepatic glycogen

Compared to the control, the LCT-treated group revealed a highly significant reduction in the hepatic glycogen content by 1.6-fold (Table 5).

**Table 5:** Hepatic glycogen, pyruvate, and lactate in experimental groups.

Parameter	Treatment group (n = 10)		F- value of the one-way ANOVA
	Control	LCT	
Glycogen (mg/g)	12.99 ± 0. 73	7.74 ± 0.26*	456.23
Pyruvate (umol/g)	0.18 ± 0.03	0.09 ± 0.02*	310.85
Lactate (umol/g)	2.35 ± 0.25	4.23 ± 0.27*	265.28

*LCT* lambda-cyhalothrin.

*p < 0.001 when values are compared to control group.

Values are expressed as mean ± SD.

n = number of rats per group.

### Hepatic lactate and pyruvate

Contrary to the significant reduction in the pyruvate concentrations by twofold, the LTC group revealed a highly significant elevation in the lactate concentrations by 1.8-fold, compared to the control group (Table 5).

### Histopathology

**Figure 1:**
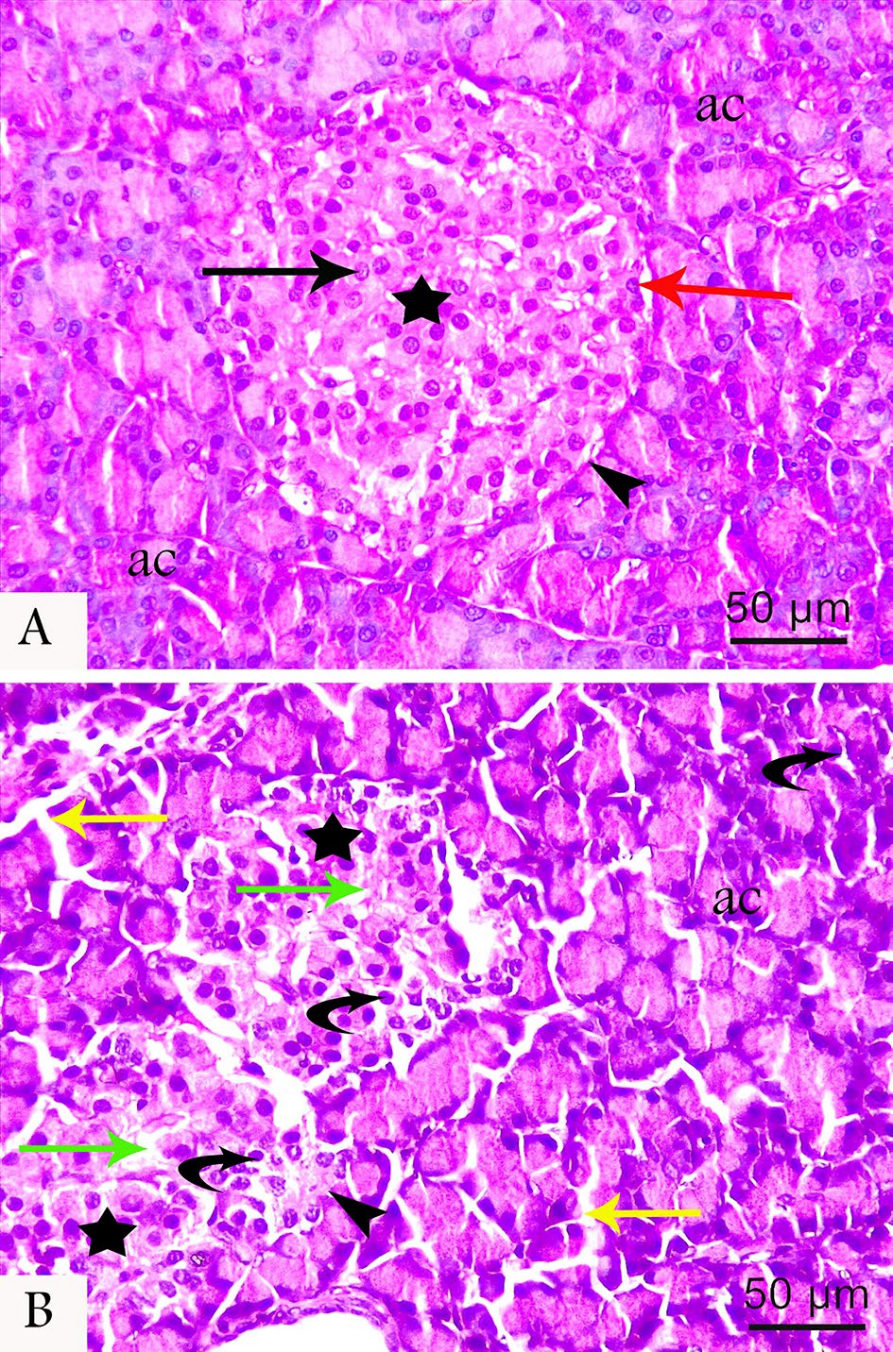
Photomicrographs of H&E stained pancreatic sections at (400 × magnification) from the control (**A**) and LCT (**B**) groups. Control group shows normal architecture in the form of regular islet of Langerhans (star) surrounded by a well-defined border (arrowhead) and distributed in between the regular closely packed acini (ac), with cords of polygonal β-cells (black arrow) in the centre and α-cells at the periphery (red arrow). In LCT group, islets show degenerative changes in the form of irregular indistinct borders (arrowhead), β-cells loss (green arrow), and pyknotic nuclei surrounded by vacuolated cytoplasm (curved arrow) in cells of islets (star) and acini (ac). Notice the diminished islets size, and the wide interlobular septa (yellow arrow) in the LCT-treated group.

H&E stained sections of control group demonstrated normal structure of pancreas as presented by regular, well-defined, lightly stained islets of Langerhans. The islets between the regularly closely packed acini were lined by pyramidal cells with apical acidophilic granules and basal basophilia. The islet cells appeared as cords of cells with intervening blood capillaries. Within the islets core, β-cells appeared as polygonal cells with pale acidophilic cytoplasm and central vesicular nuclei while α-cells were noticed as smaller cells with small, dark nuclei at the periphery (Fig. 1A). In the LCT group, islets revealed variable degenerative changes including reduced islets size, irregular indistinct borders, diminished cell density, pyknotic nuclei, vacuolated cytoplasm, and β-cells loss. Moreover, acini showed degenerative changes in the form of cytoplasmic vacuolations in cells with widened interlobular spaces (Fig. 1B).

**Figure 2:**
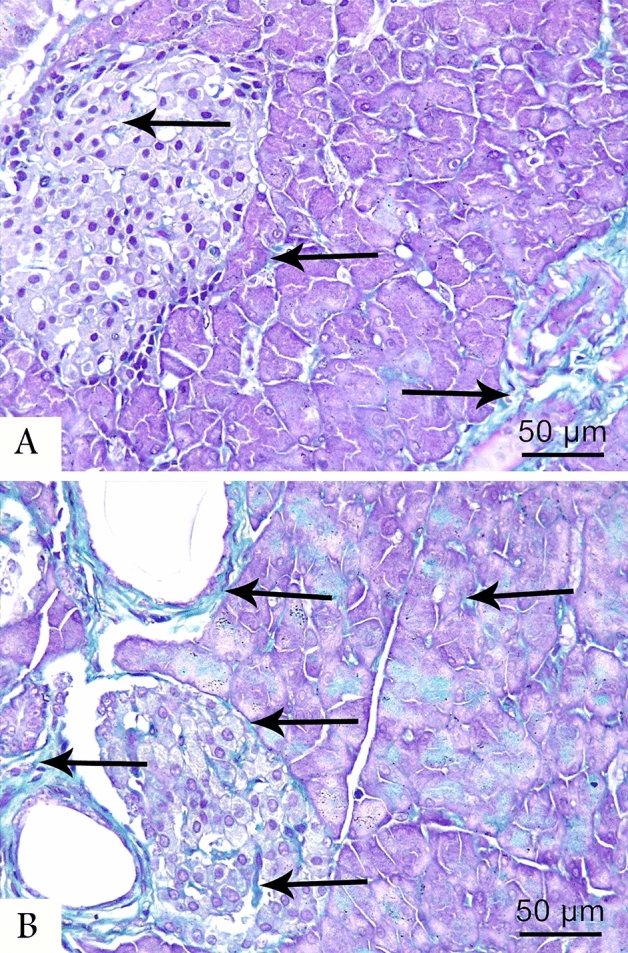
Photomicrographs of Masson’s trichrome stained pancreatic sections at (400 × magnification) from the control (**A**) and LCT (**B**) groups showing normal distribution of collagen fibers (arrow) in the septa and around the acini and blood capillaries of control group. In LCT group, dense collagen fibers (arrow) are observed around some pancreatic ducts, blood vessels, within the endocrine cells of the islets, and around the blood capillaries.

Masson’s trichrome stained pancreatic tissues showed normal distribution of the collagen fibers in control group, appearing as delicate fibers in the septa and around the acini and blood capillaries (Fig. 2A). However, in the LCT-treated group, dense connective tissue fibers were noticed around some pancreatic ducts and blood vessels, within the endocrine cells of islets of Langerhans, and around blood capillaries (Fig. 2B).

### Immunohistochemistry

**Figure 3:**
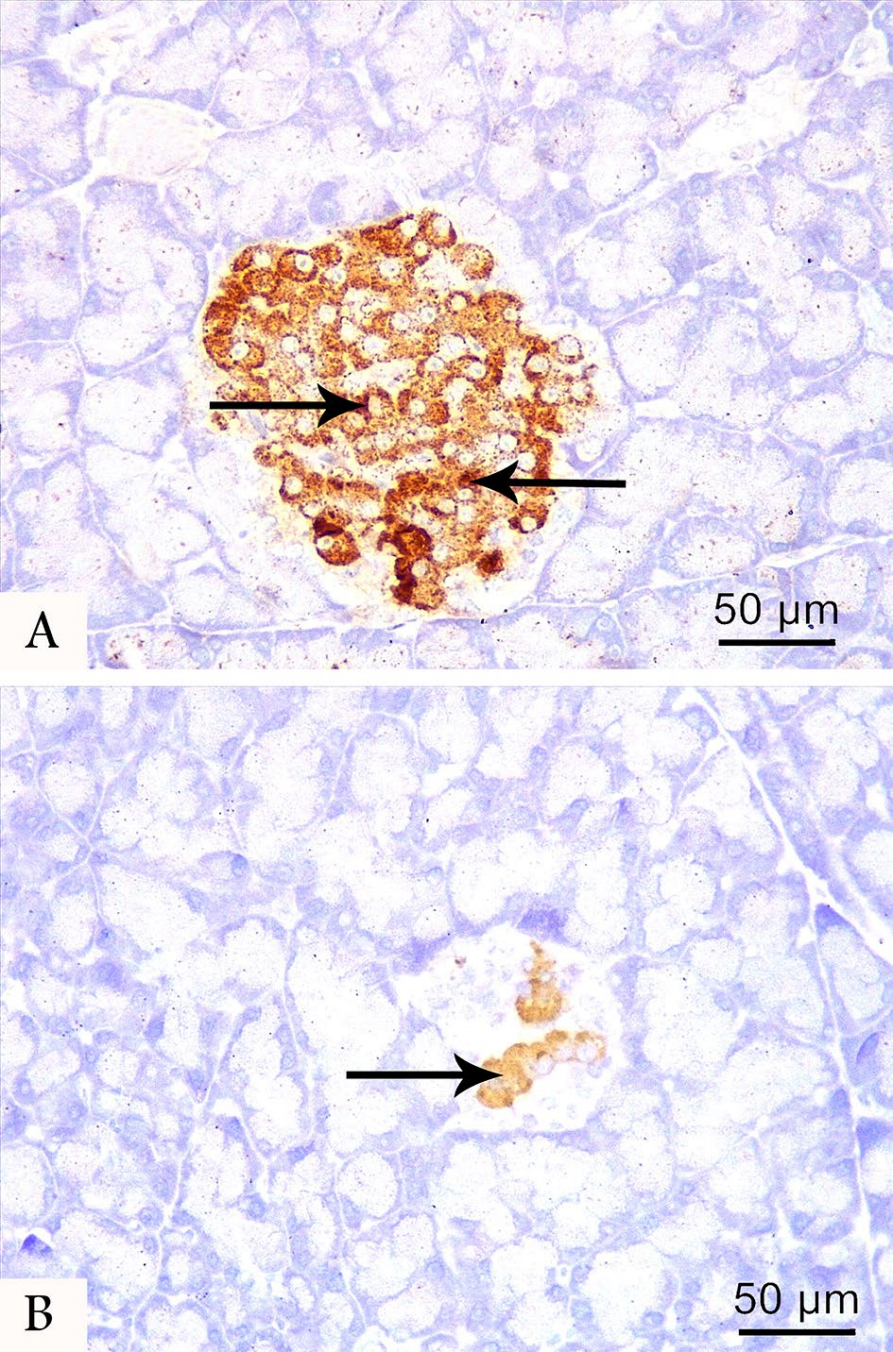
Photomicrographs of anti-insulin immunostained pancreatic sections at (400 × magnification) from the control (**A**) and LCT (**B**) groups showing strong cytoplasmic immunoreactivity (arrow) in most of β-cells of the control group and weak immunoreaction in few β-cells of LCT group.

**Figure 4:**
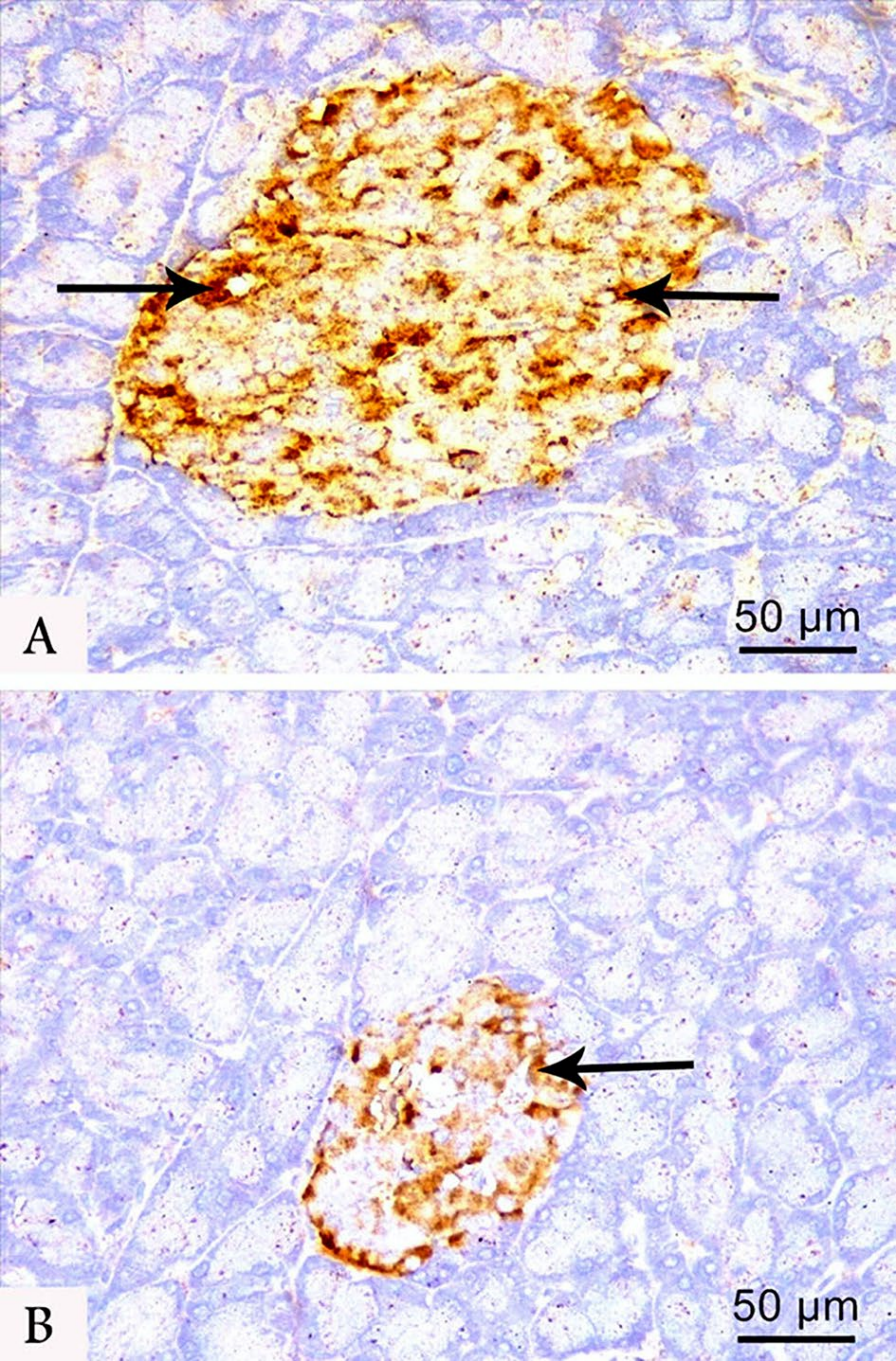
Photomicrographs of anti Nrf2 immunostained pancreatic sections at (400 × magnification) from the control (**A**) and LCT (**B**) groups showing strong cytoplasmic immunoreactivity (arrow) to Nrf2 in most of islet cells of the control group and weak positive reaction in LCT group.

**Figure 5:**
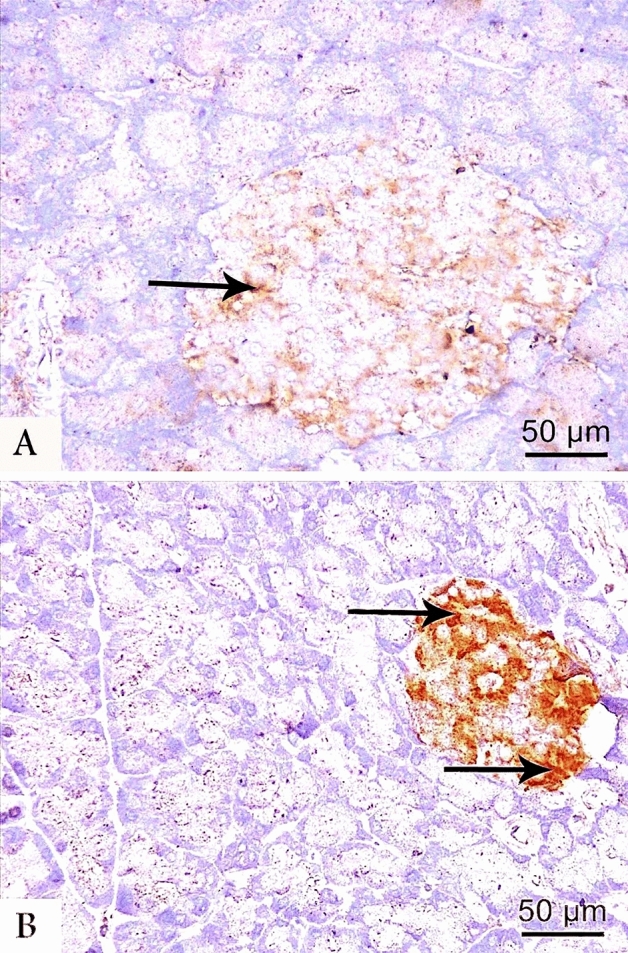
Photomicrographs of anti TNF-α immunostained pancreatic sections at (400 × magnification) from the control (**A**) and LCT (**B**) groups showing weak immunoreactivity (arrow) in the control group and strong cytoplasmic immunoreaction to TNF-α in most of cells in LCT group**.**

In the control group, the immunohistochemical localization of insulin-secreting β-cells demonstrated a markedly enlarged positive β-cells core in islets of Langerhans (Fig. 3A). Meanwhile, a weak insulin immunoreactivity was detected in few numbers of β-cells within the shrunken islets of the LCT group (Fig. 3B). Additionally, the control group demonstrated a strong cytoplasmic immunoreactivity to Nrf2 in most islet cells, while weak positive reaction was noticed in a few islet cells in LCT-treated group (Fig. 4A,B). On the other hand, the control group displayed a weak reaction to TNF-α. By the contrary, a strong cytoplasmic reaction to TNF-α was noticed in most cells of LCT-treated rats (Fig. 5A,B).

The morphometric analyses of LCT group showed a highly significant reduction in the mean diameter of islets of Langerhans by 1.4-fold and in area % of both insulin and Nrf2 immunoreaction by 3.8 and 1.6-fold, respectively. Meanwhile, a highly significant increase in the area % of collagen fibers by 1.7-fold and TNF-α by twofold was detected in the LCT group when compared to the control (Table 6).

**Table 6:** Morphometric parameters of pancreas in experimental groups.

Parameter	Treatment group (n = 10)		F- value of the one-way ANOVA
	Control	LCT	
Diameter of islets (um)	136.90 ± 8.13	95.40 ± 4.03*	208.954
Collagen fibers (Area % of collagen /HPF)	11.51 ± 0.24	20.26 ± 0.43*	3170.775
Insulin (Area % of positive cells /HPF)	10.87 ± 0.65	2.83 ± 0.14*	1481.824
Nrf2 (% of positive cells/HPF)	10.02 ± 0.60	5.95 ± 0.20*	417.628
TNF-α (% of positive cells/HPF)	8.01 ± 0.48	16.34 ± 0.56*	1286.810

*LCT* lambda-cyhalothrin, *Nrf2* nuclear factor erythroid 2–related factor 2, *TNF- α* tumor necrosis factor-alpha, *HPF* high power field.

*p < 0.001 when values are compared to control group.

Values are expressed as mean ± SD.

n = number of rats per group.

## Discussion

The LCT-treated group revealed a highly significant elevation in serum glucose levels and reduction in insulin levels. Consistent with our results, oral exposure to low (1/40 of LD50) and high (1/4 of LD50) doses of LCT-for 3 months-in rats has resulted in significant hyperglycemia and hypoinsulinemia throughout the experiment (after 1st, 2nd and 3rd month of treatment periods)^32^. Moreover, administration of LCT in rabbits has significantly increased blood glucose levels^33^. Similarly, 60-day DLM administration has shown significant rise in blood glucose along with apparent decrease in insulin levels in rats^15^. Also, Indian major carp Labeo rohita^34^ has also exhibited CYP-induced hyperglycemia.

Despite being non-specific, hyperglycemia stands for a quick response to the toxicity of type II pyrethroids^35^. Many explanations have been suggested to explain hyperglycemia. Pyrethroids trigger a state of physiological stress which stimulates the sympathetic nervous system to release catecholamine and glucocorticoid, causing hyperglycemia^32,36^. Additionally, pyrethroids enhance a state of sustained neuronal discharge, which stimulates glucose production by gluconeogenesis and glycogenolysis in compensation for the increased energy demand^37^.

Oxidative stress is the primary mechanism for LCT toxicity^5,32^. Unfortunately, β-cells of the pancreas are especially liable to oxidative stress because of the excess production of intrinsic reactive oxygen species (ROS) and defective expression of antioxidant enzymes like SOD, glutathione peroxidase (GPx), and CAT^38^. At molecular level, the presence of mitochondria within membranes, renders them as the most preferable targets for the pyrethroids-induced toxicity^39^.

Oxidative injury of β-cells impairs mitochondrial ability to link glucose stimulus to insulin secretion in response to hyperglycemia, resulting in defective insulin production^40^. Moreover, the excessive production of ROS further aggravates the pyrethroids induced-mitochondrial dysfunction via enhancing the endoplasmic reticulum (ER) stress. Subsequently, ER stress alters Ca2 + homeostasis in mitochondria leading to increased mitochondria dysfunction, excess ROS generation, and eventually β-cells exhaustion and loss^39^. In two recent studies, oxidative stress has been perceived as a primary cause for β-cells dysfunction and loss in the pancreas of the LCT^32^ and DMT^15^- treated rats.

In same line, results revealed a highly significant increase in MDA levels along with decreased endogenous antioxidants (CAT, SOD, and GSH) in LCT group. LCT has been proved to enhance lipid peroxidation and disrupt redox balance in various tissues, via ROS generation^9,41^. Interestingly, LCT does not produce free radicals directly, but it liberates different radicals indirectly upon decomposition into cyanides and aldehydes^11^. Also, LCT may possess a direct toxic effect on the antioxidant enzymes^42^. Consistent with our results, LCT increased MDA levels in kidneys of rats^4^, kidneys and livers of mice^43^, and blood of rats^5^. On the other hand, LCT decreased CAT, SOD, and GSH levels in various tissues^4,19^. Also, DMT resulted in lipid peroxidation and impaired antioxidants (CAT, SOD, GPx, and GSH) in the pancreas of treated rats^15^.

In conjunction with the altered redox balance, the protein levels of Nrf2 and its downstream HO-1 were significantly reduced in LCT group. Nrf2 is the master regulator of the antioxidant response element (ARE)-driven gene expression, which encodes phase II detoxifying enzymes like HO-1 and NADPH quinone oxidoreductase 1 (NQO-1); the antioxidant enzymes like CAT, SOD, and GPx; and the non-enzymatic antioxidants like GSH^44^. In pancreas, Nrf2 protects β-cells against diabetic stresses via amelioration of oxidative stress, maintenance of β-cells mass, preservation of insulin content and secretion, and regulation of mitochondrial function and biogenesis^45^.

However, the protective effect mediated by Nrf2 pathway is guided by the dose and duration of exposure to the toxin. Thus, repeated exposure to a toxin restrains the Nrf2 signaling pathway in tissues and restricts phase II detoxifying enzymes activation^46^. Furthermore, Nrf2 dysfunction impairs β-cells proliferation and decreases insulin production by the pancreas, resulting in hyperglycemia^45^. Consistent with our results, in rats administered DLM for 21 days, the outflow of Nrf2/ HO‐1 pathway was markedly diminished in kidney^47^. Also, in rats treated with CYP for 24 and 48 h, the expression of Nrf2, HO-1, and NQO1 genes was sharply downregulated in association with reduced antioxidants^46^.

In the current study, the significant elevation in pancreatic TNF-α, IL-6, and NF-κB levels was indicative of the inflammatory response caused by LCT in the pancreas. The NF-κB is a redox-regulated transcriptional factor controlling inflammation and cellular injury. Excess ROS permits NF-κB pathway activation and the subsequent upregulation of genes of the pro-inflammatory cytokines (TNF-α, IL-6)^48^. The NF-κB pathway is negatively controlled by Nrf2. Consequently, downregulation of Nrf2/HO-1 pathway activates NF-κB pathway, via oxidative mechanism^48^.

In line with our results, rats administered 0.6 mg/kg b.wt. (1/100 of LD_50_ LCT) for 10 weeks have demonstrated increased NF-ĸB/p65 and pro-inflammatory protein levels, in addition to increased inflammatory cytokines^9^. Also, short term exposure to LCT at two doses (4 mg/kg and 8 mg/kg b.wt.) for 6 days has resulted in increased expression of NF-κB and IL-1β in livers of treated rats^49^. Furthermore, TNFα, IL-1β, IL-6 and INF-γ have significantly elevated in serum^9^ and liver^50^ of CYP-treated rats secondary to NF-ĸB pathway activation, via oxidative stress mechanism^50^.

The pancreas plays the most crucial role in directing the hepatic glucose metabolism via insulin secretion^51^. Therefore, we have investigated the activity of hepatic enzymes of glucose metabolism. As expected by the literature, our findings revealed a clear disturbance in the glucose metabolism, as evidenced by the reduced activity of hexokinase and glycogen synthase enzymes, and the increased activity of glucose-6-phosphatase, lactate dehydrogenase, and glycogen phosphorylase in LCT-treated rats.

Hexokinase enzyme is the gateway for glucose in glycolysis. It is an absolute insulin sensitive and dependent enzyme and almost inhibited in insulin-deficient rats^52^. On the other hand, glucose-6-phosphatase is one of two enzymes mediating gluconeogenesis. Decreased hexokinase activity and increased glucose-6-phosphatase are mainly attributed to the insulin deficiency^17^. Similarly, the diminished hexokinase and increased glucose-6-phosphatase activity have been ascribed to the insulin deficiency in the DLM**-**treated rats^15^.

Glycogen synthase and glycogen phosphorylase are the two key rate-limiting enzymes regulating the glycogen metabolism^51^. Insulin stimulates glycogen synthesis via activation of protein phosphatase 1 and inhibition of kinases like protein kinase A and glycogen synthase kinase 3^53^. Accordingly, decreased insulin secretion reduces glycogen content^54^. Depletion of hepatic glycogen has been previously reported in CYP-treated Clarias batrachus^54^ and DMT-treated rats^15^.

Additionally, our results suggested increased anaerobic glycolysis in the liver as reflected by the raised lactate dehydrogenase activity, excess lactate production, and reduced pyruvate concentration in the LCT group. LDH is the main enzyme of anaerobic glycolysis, which catalyzes the irreversible interconversion of pyruvate to lactate under anaerobic conditions^34^. Pyrethroids have been reported to favor a hypoxic condition in liver lobules and increase the lactate dehydrogenase activity^34^. Parallel to our results, LDH activity has markedly increased in fish exposed to LCT^55^ and CYP^34,55^. Moreover, increased lactate and decreased pyruvate levels have been reported in muscle, gill, and liver of Labeo rohita exposed to a sub-lethal dose of DMT^56^.

In the present study, Histopathological examination and immunohistochemical results collaborated with the results of biochemistry. Sections of the LCT-treated group revealed variable degenerative changes, including reduced islet size, decreased cellularity, and β-cells loss. These results were further supported by Elhalwagy et al.^32^ and Feriani et al.^15^ who noted atrophy of islets, disturbed acinar architecture, and distorted pancreatic ducts. Tuzmen et al.^57^ attributed the degenerative changes in the pancreas to the oxidative stress injury. Also, Elhalwagy et al.^32^ ascribed the cell wall damage and the impaired depolarization to the oxidative stress and the reduced free radical scavengers.

Masson’s trichrome stain revealed a significant rise in the area % of collagen fibers in LCT group, which copes with Abdul-Hamid et al.^19^ who noticed excessive accumulation of collagen in liver of LCT-treated rats. Oxidative stress is also implicated in the fibrotic changes detected in the pancreas, mostly via pancreatic stellate cells activation, which invade the islets causing fibrosis^58^.

In the LCT-treated group, reduced serum insulin was further supported by the decreased area % of insulin expression. The insufficient secretion of insulin was ascribed to the oxidative stress-induced mitochondrial dysfunction and β-cells damage^15^. Additionally, the reduced area % of Nrf2 immunoreactivity and the increased expression of TNF-α in the LCT group were consistent with the detected alterations in Nrf2, HO-1, and NF-κB protein levels. The depletion of Nrf2 impedes β-cells survival, proliferation, and insulin secretion^45^. Meanwhile, increased TNF-α expression enhances induction and progression of inflammatory response^49^.

## Conclusion

Subacute exposure to LCT resulted in pancreatic toxicity as reflected by the diminished insulin secretion and β-cells loss. Pancreatic toxicity was mostly attributed to oxidative and inflammatory mechanisms. Oxidative stress was indicated by the reduced Nrf2 and HO-1, along with the diminished antioxidant enzymes. The increased NF-κB and excess inflammatory cytokines were indicative of the inflammatory response. Correspondingly, LCT dysregulated insulin dependent enzymes of glucose metabolism, resulting in hyperglycemia, decreased pyruvate, and increased lactate concentrations. Therefore, routine diabetes screening tests are warranted for the LCT-exposed workers. Also, further studies are required to investigate the potential protective effects of natural antioxidants and anti-inflammatory agents against LCT-induced pancreatic toxicity.

## Data Availability

The datasets generated and analyzed during the study are available from the corresponding author on reasonable request.
